# Supplementary material for: A Likelihood Approach for Real-Time Calibration of Stochastic Compartmental Epidemic Models
Source: PLoS Comput Biol. 2017 Jan 17;13(1):e1005257. doi: 10.1371/journal.pcbi.1005257 (PMC5240920; doi:10.1371/journal.pcbi.1005257)
Supplement: S1 File — (TAR.GZ) [file pcbi.1005257.s014.tar.gz › HSPH_Online-SI-Revision/MSS10/n100-severe/n100-severe_NEW2_summary2.pdf]

peak -8	3w spec	3w cum	5w spec	5w cum	end	3w spec LOG	3w cum LOG	5w spec LOG	5w cum LOG	end LOG
mean	375.2	337.1	105.5	161.1	35.5	0.5	0.4	0.5	0.4	0.3
median	66.6	60.	75.8	70.5	36.	0.4	0.3	0.4	0.4	0.2
quantile .1	19.8	19.2	15.6	16.4	20.	0.1	0.1	0.1	0.1	0.1
quantile .9	971.7	785.9	252.5	440.7	51.6	1.1	0.9	1.2	0.1	0.6
35	37	36	37	25						

peak -4	3w spec	3w cum	5w spec	5w cum	end	3w spec LOG	3w cum LOG	5w spec LOG	5w cum LOG	end LOG
mean	19.3	16.6	32.6	18.3	31.4	0.1	0.1	0.2	0.1	0.2
median	19.3	16.	34.5	15.9	30.7	0.1	0.1	0.2	0.1	0.1
quantile .1	7.5	6.2	10.3	5.9	11.3	0.	0.	0.	0.	0.
quantile .9	29.9	27.3	53.9	32.1	51.5	0.2	0.1	0.3	0.	0.3
22	26	16	16	13						

peak	3w spec	3w cum	5w spec	5w cum	end	3w spec LOG	3w cum LOG	5w spec LOG	5w cum LOG	end LOG
mean	20.	12.1	30.3	15.	18.4	0.1	0.1	0.2	0.1	0.1
median	18.9	10.8	31.	13.	14.4	0.1	0.	0.1	0.1	0.1
quantile .1	6.4	3.	8.4	3.5	3.8	0.	0.	0.	0.	0.
quantile .9	32.5	22.6	47.2	27.7	35.	0.2	0.1	0.3	0.	0.2
5	8	8	6	4						

peak +4	3w spec	3w cum	5w spec	5w cum	end	3w spec LOG	3w cum LOG	5w spec LOG	5w cum LOG	end LOG
mean	20.9	13.5	40.4	14.8	16.2	0.1	0.1	0.2	0.1	0.1
median	19.9	12.9	22.6	13.5	14.3	0.1	0.1	0.1	0.1	0.1
quantile .1	5.5	5.3	8.4	5.1	5.4	0.	0.	0.	0.	0.
quantile .9	35.1	20.4	57.2	23.3	28.5	0.2	0.1	0.3	0.	0.1
17	18	28	18	17						

peak +8	3w spec	3w cum	5w spec	5w cum	end	3w spec LOG	3w cum LOG	5w spec LOG	5w cum LOG	end LOG
mean	380.9	57.4	209.8	59.6	60.5	0.3	0.2	0.4	0.2	0.2
median	47.9	33.6	60.2	32.7	29.3	0.2	0.2	0.3	0.2	0.2
quantile .1	16.2	9.4	20.2	9.9	11.6	0.1	0.	0.1	0.	0.1
quantile .9	213.7	114.2	564.	121.5	119.3	0.7	0.4	0.8	0.	0.4
23	20	28	20	21	{}	{}	{}	{}	{}	{}
